# Correspondence

**Published:** 1874-08

**Authors:** J. D. Rush

**Affiliations:** Vernon, Ala., Sanford county


VERNON, ALA., Sanford county, May 12, 1874.

*To the Editors of the Record:*

I send to you for publication a case of direct fracture of the os-frontis. A little boy of Mr. B.'s, some eight years old, produced by a kick from a mule, on the 18th of December, 1873. Dr. M. W. Morton and myself were called to the case in great haste, the distance being some eight miles. A space of some hours elapsed from the time of the accident until we saw the boy. We found him having convulsions and speechless. A full dose of chloral hydrate was given at once, and in a short interval stimulants were administered until a normal circulation was restored, after which he was put under the influence of chloroform. On making an examination, we found that the bone was cut loose from the inner to the outer angle of the eye, in a semi-circular form, and forced in upon the brain. The blow having made such a complete cut of the part that no dissecting was necessary in the case. Having no trephine with us, we devised an instrument with a slight curve at the end, which acted as a hook. With this we succeeded in elevating the depressed bone, by first removing a small piece of the nasal tuberosity. The wound was not closed at all, more than the natural adjustment of the parts, which seemed to be quite sufficient. After the operation, there being some symptoms of convulsions, he was put on full doses of potass. bromide, every three or four hours, until these symptoms subsided, and at the same time controlled the arterial excitement with veratrum viride. Bowels kept in soluble condition. The wound healed by union of the second intention, during which time several small spiculæ of bone worked out. The The boy is well and hardy. Very Respectfullly,

J. D. RUSH, M.D.

